# A Multi-Pumping Flow System for In Situ Measurements of Dissolved Manganese in Aquatic Systems

**DOI:** 10.3390/s16122027

**Published:** 2016-11-30

**Authors:** David Meyer, Ralf D. Prien, Olaf Dellwig, Joanna J. Waniek, Ingo Schuffenhauer, Jan Donath, Siegfried Krüger, Malte Pallentin, Detlef E. Schulz-Bull

**Affiliations:** Leibniz Institute for Baltic Sea Research Warnemünde, Seestr. 15, 18119 Rostock, Germany; ralf.prien@io-warnemuende.de (R.D.P.); olaf.dellwig@io-warnemuende.de (O.D.); joanna.waniek@io-warnemuende.de (J.J.W.); ingo.schuffenhauer@io-warnemuende.de (I.S.); jan.donath@io-warnemuende.de (J.D.); siegfried.krueger@io-warnemuende.de (S.K.); malte.pallentin@io-warnemuende.de (M.P.); detlef.schulz-bull@io-warnemuende.de (D.E.S.-B.)

**Keywords:** manganese, in situ, multi-pumping flow system, sea water, redoxcline, Baltic Sea

## Abstract

A METals In Situ analyzer (METIS) has been used to determine dissolved manganese (II) concentrations in the subhalocline waters of the Gotland Deep (central Baltic Sea). High-resolution in situ measurements of total dissolved Mn were obtained in near real-time by spectrophotometry using 1-(2-pyridylazo)-2-naphthol (PAN). PAN is a complexing agent of dissolved Mn and forms a wine-red complex with a maximum absorbance at a wavelength of 562 nm. Results are presented together with ancillary temperature, salinity, and dissolved O${}_{2}$ data. Lab calibration of the analyzer was performed in a pressure testing tank. A detection limit of 77 nM was obtained. For validation purposes, discrete water samples were taken by using a pump-CTD system. Dissolved Mn in these samples was determined by an independent laboratory based method (inductively coupled plasma–optical emission spectrometry, ICP-OES). Mn measurements from both METIS and ICP-OES analysis were in good agreement. The results showed that the in situ analysis of dissolved Mn is a powerful technique reducing dependencies on heavy and expensive equipment (pump-CTD system, ICP-OES) and is also cost and time effective.

## 1. Introduction

Manganese (Mn) is the second most abundant transition metal in the Earth’s crust after iron (Fe), and is widely distributed in nature [1]. Like Fe, it exhibits a multitude of biogeochemical reactions due to its empty and partially occupied d-orbitals, which enable it to bond reversibly to many functional groups. Thus, Mn is highly reactive and therefore is involved in a variety of processes in the marine environment.

Mn plays an important and complex role in the ocean, and thus remains at the focus of attention for many scientists. It belongs to the group of essential trace elements, and hence is assimilated by all living organisms. The averaged cellular Mn concentration of phytoplankton, for example, is 420 mmol/L [2]. Moreover, Mn is of critical importance for many metabolic and non-metabolic regulatory functions [3], and is highly relevant in the detoxification of free radicals [4]. In remote oceanic regimes with high nutrients and low atmospheric input, Mn could even potentially limit oceanic productivity [5,6]. Furthermore, it has been demonstrated that there is a tight link between Mn cycling and numerous other elements, such as oxygen, nitrogen, sulfur, phosphorus, and several trace metals, like iron, lead, and copper [7,8,9,10,11]. In particular, the coupling of redox transformations and transport processes taking place across redox boundaries in sediments has been a subject of many studies [12,13,14,15], but pelagic oxic/anoxic boundaries (redoxcline) of stratified basins were also studied extensively during the last decades [10,16,17,18,19,20,21,22].

In general, Mn exists in seawater in three oxidation states (II, III, and IV), with Mn(II) and Mn(IV) being the most prevalent species. According to the oxidation state, the physico-biochemical behavior of Mn changes, as the oxidation state determines the solubility properties, and hence the bioavailability. In oxygenated seawater, particulate Mn(IV)-oxides, -hydroxides, and -oxyhydroxides are the most thermodynamically stable forms of Mn, whereas in conditions of low p$O_{2}$, dissolved Mn(II) is the dominant species. In addition, dissolved Mn(III) has been found to be an important intermediate of Mn(II) oxidation [23] and Mn(IV) reduction [24], and was recently determined in the pelagic redoxcline of the Black and Baltic Seas [19,21] and in pore waters of the St. Lawrence estuary [25].

Thus, the determination of dissolved Mn is of considerable interest for studies in marine biogeochemistry, and is essential for understanding and interpreting the chemical reactivity of Mn in nature. With regard to the proposed rapid and substantial change the ocean ecosystem is facing due to global climate change (i.e., ocean deoxygenation, acidification, and temperature increase), extending knowledge about dissolved Mn may also be relevant for modeling studies, in order to accurately describe the alteration this redox-sensitive element undergoes in the future [26,27,28].

Since the distribution of redox sensitive species such as Mn can strongly vary at oxic/anoxic boundaries in space and time, conventional discrete sampling approaches are usually not very well suited for resolving small-scale and short-term variability. Special equipment, such as a pump-CTD is needed to match the requirements of small-scale sampling. Samples can then be analyzed on-shore using laboratory based methods (e.g., inductively coupled plasma optical emission spectrometry, ICP-OES). Another, less elaborate, method to get small-scale and short-term variability is through the use of in situ analyzers, which is more time and cost effective, provided that the accuracy and sensitivity of the used analyzers are sufficient for the detection of Mn. They allow rapid analyses directly at the location of interest, and thus avoid sample handling and storage. In recent years, new methods for the determination of dissolved Mn using in situ analyzers have been developed, including fluorescence [29], chemiluminescence [30], voltammetry [31,32], and spectrophotometry [33,34]. Mn analyzers have been fitted to autonomous underwater vehicles (AUVs), remotely operated vehicles (ROVs), submarine cables, and CTD profiling packages, and were used, for instance, in fjords and hydrothermal environments [6,33,35,36,37,38].

Several analyzer systems that can be used for the in situ determination of dissolved Mn are reported in the literature, such as SCANNER [39], SUAVE [40], GAMOS [41], and certain microelectrodes [42], as well as a novel microfluidic system [43]. Although all of them were successfully deployed in the field, most of them did not possess all of the features needed to fully resolve small-scale variability of dissolved Mn at low micromolar levels as they occur, for example, across the pelagic redoxclines of the central Baltic Sea basins. GAMOS has a response time of approximately 6 min. [44] and other systems only showed measurement frequencies of 10 s [37] and 8 min [43], respectively. Microelectrodes, however, have a too-high minimum detection limit of about 3 μM–5 μM [42] and in general have a relatively short deployment time.

In this study, we use a modified version of the METals In Situ analyzer system METIS, developed by Prien et al. [45]. The long-term goal is to equip a profiling mooring (GODESS; Gotland Deep Environmental Sampling Station)—which is already successfully deployed in the central Baltic Sea [46]—with the METIS analyzer, enabling improved Mn observations over longer time periods and without ship-based sampling. This paper shows the first results of the in situ detection of dissolved Mn using the METIS analyzer in the Gotland Basin, Baltic Sea.

We adapted the spectrophotometric 1-(2-pyridylazo)-2-naphthol (PAN) method of the SCANNER system [33], due to its fast color development of less than 1 s and its efficiency, which has already been proven in aquatic environments to determine in situ low concentrations of Mn [35]. Furthermore, we used a multi-pumping flow system working with solenoid pumps and a custom-built 3-D printed mixing device to ensure high sample flow rates, and fast mixing. The combination of high flow rates, fast mixing, and a fast color development of the spectrophotometric system ultimately allows data gathering with a high vertical resolution while performing a hydrocast. A Deep Sea Telemetry system integrated into the CTD rosette sampler enabled near-real time measurements and online analyzer control.

This work presents the first multi-pumping flow-system to use the PAN method for determination of dissolved Mn concentrations at low micromolar levels and with high vertical resolution.

## 2. Materials and Procedures

### 2.1. Apparatus

**Figure 1:**
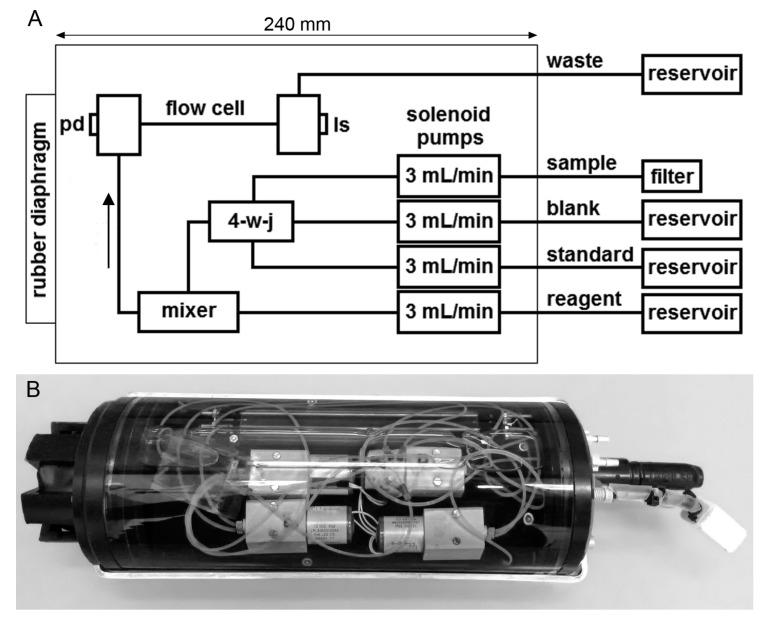
(**A**) Multi-pumping flow system manifold used for the analysis of dissolved Mn. 4-w-j: 4-way junction; ls: light source; pd: photodiode; (**B**) Photo of the METIS analyzer including the coarse sandstone filter for removing large particles.

The METIS analyzer system (240 mm length; 96 mm maximum diameter) is illustrated in Figure 1. It consists of an oil-filled acrylic tube containing the optical absorption cell (borosilicate-glass; 140 mm length; 0.8 mm i.d.; internal volume 0.07 mL; Hilgenberg GmbH, Malsfeld, Germany), the fluid handling system, four solenoid driven pumps (Lee Hydraulische Miniaturkomponenten GmbH, Sulzbach, Germany; flow rate 0.05 mL/s), a custom built 3D-printed PMMA (poly(methyl methacrylate)) mixer, and two electronic modules. All tubing connections were made with PTFE tubing (0.8 mm i.d., Bohlender, Grünsfeld, Germany) and Lee MINSTAC fittings (Lee Hydraulische Miniaturkomponenten GmbH, Sulzbach, Germany). A rubber diaphragm integrated in the bottom of the tube allows for pressure compensation. The silicon oil chosen (Quax GmbH, Otzberg, Germany) showed a viscosity of 10 mm${}^{2}$/s and a viscosity/temperature coefficient of 0.56, and thus did not decrease the flow rates of the pumps at low water temperatures. METIS is a compact, versatile, microcontroller-based measurement and control system using the Sensor Group Data Logger (SGDL) developed in the Sensors Group at the National Oceanography Centre Southampton (NOCS). A dedicated analyzer software was written in C using the data logger’s application programming interface, allowing the user to change pumping speed or detector integration time before the deployment. The pump cycles of all pumps were set to 1 s. The reagent pump was activated during the entire hydrocast, and the other pumps were activated as needed with a delay of 0.5 s relative to the reagent pump, so that at any given time, two pumps were working. Since the non-working pumps act as closed valves, there was no need for using additional valves in the analyzer. Thus, material costs and the vulnerability to failures of the system was kept low. The resulting flow rates of the activated streams were 3 mL/min before and 6 mL/min after reaching the mixing device. No significant back flow from the mixer to the different reservoirs could be observed. Electrical power can be supplied in two different ways. METIS can either be powered via the interface of the telemetry system (SST, Trappenkamp, Germany) or by a custom-made 24 V, 15 Ah alkaline battery pack (16× Duracell ProCell MN 1300 LR20, RS Components GmbH, Mörfelden-Walldorf, Germany). In this study, we used the telemetry system of the pump-CTD, because it allowed online control of the analyzer.

The analytical reagents were stored outside the analyzer in flexible plastic bags made out of ethylene vinyl acetate (HEMEDIS GmbH, Weißenborn, Germany). The sample stream was filtered through a coarse sandstone-filter (Schego, Offenbach am Main, Germany) with a nominal cut-off of 10 μm to remove large particles. In order to prevent possible contamination, the filter was pre-cleaned with 2 vol % sub-boiled HNO${}_{3}$ just before its use. The sample fluid was then mixed at a 1:1 ratio with the reagent solution, and was passed through the absorption cell. A custom-built mixer was used for mixing purposes, instead of a coil of tubing. The length of tubing between mixer and flow cell was 25 cm, and the length of tubing between sample inlet and mixer was 62 cm, respectively. A LED (TLPGE18TP(F), Toshiba America Electronic Components Inc., Irvine, CA, USA) with a peak emission wavelength of 562 nm was used as a light source, and a photodiode with a light to frequency converter (TSL237, AMS-TAOS USA Inc., Plano, TX, USA) was used as a detector (integration interval 1000 ms). Detected frequencies are proportional to the amount of light passing the absorption cell. Thus, low frequency values correspond to high absorbance values, and vice versa. Results were automatically written to an SD-card storing information about detector output, time, and activated pumps. A rosette sampler was equipped with 10 Free-Flow bottles (Hydro-Bios, Kiel, Germany) and a CTD probe (Model SBE-9, Sea-Bird Electronics, Inc., Bellevue, WA, USA), which were used for ancillary chemical and physical analysis, respectively. The METIS analyzer was attached to the CTD rosette frame, and the system was deployed at a speed of about 0.1 m/s. At the end of the hydrocast, an in situ calibration was performed, measuring a blank and a standard solution.

### 2.2. Flow Cell

To ensure that the light coming from the LED cannot leave the glass capillary of the flow cell, the outer surface of the capillary was metallized by adhering a thin coat of silver according to Liebig’s method [47]. For this purpose, a silver nitrate and a glucose solution were needed.

The silver nitrate solution was prepared by the following steps: An ammonia solution (NH${}_{4}$OH, 25%) was added dropwise under stirring to 100 mL of a silver nitrate solution (AgNO${}_{3}$, 5%) until emerging precipitation (brown staining) disappeared. Ammonium sulfate (${\left({\mathrm{NH}}_{4}\right)}_{2}$SO${}_{4}$, 1.5 g) was then added and completely dissolved by further stirring in order to prevent the formation of silver nitride (Ag${}_{3}$N), which is an explosive chemical compound. Finally, the resulting solution was topped up with Milli-Q water (ultrapure water with a specific resistance ≥18 MΩ·cm${}^{-1}$ obtained by passing deionized water through a special filtering system, Merck Millipore, Bellerica, MA, USA) to a total volume of 500 mL and stored in a brown flask (borosilicate, Brand GmbH + Co KG, Wertheim, Germany). The glucose solution was prepared as follows: First, 4.5 g of potassium hydroxide (KOH) were completely dissolved in 150 mL Milli-Q water. Second, 1.8 g of glucose were added, and the resulting solution was also topped up with Milli-Q water to a total volume of 500 mL.

Before the process of silver plating started, both ends of the capillary were flame-sealed to protect the inner cell surface. Afterwards, the solutions were warmed up and added at a 1:1 ratio to a custom built plastic vessel already containing the glass capillary. The reaction vessel was made out of plastic in order to have no other glass surfaces and thus prevent competitive reactions of silver plating. After the reaction (10 min), the capillary was carefully cleaned with Milli-Q water. To achieve a good covering of the surface, the silver plating procedure was repeated two more times. The ends of the capillary were then cut by using a ceramic cutting device. Finally, heat shrink tubing was used to protect the capillary and its coating.

### 2.3. Mixing Cell

**Figure 2:**
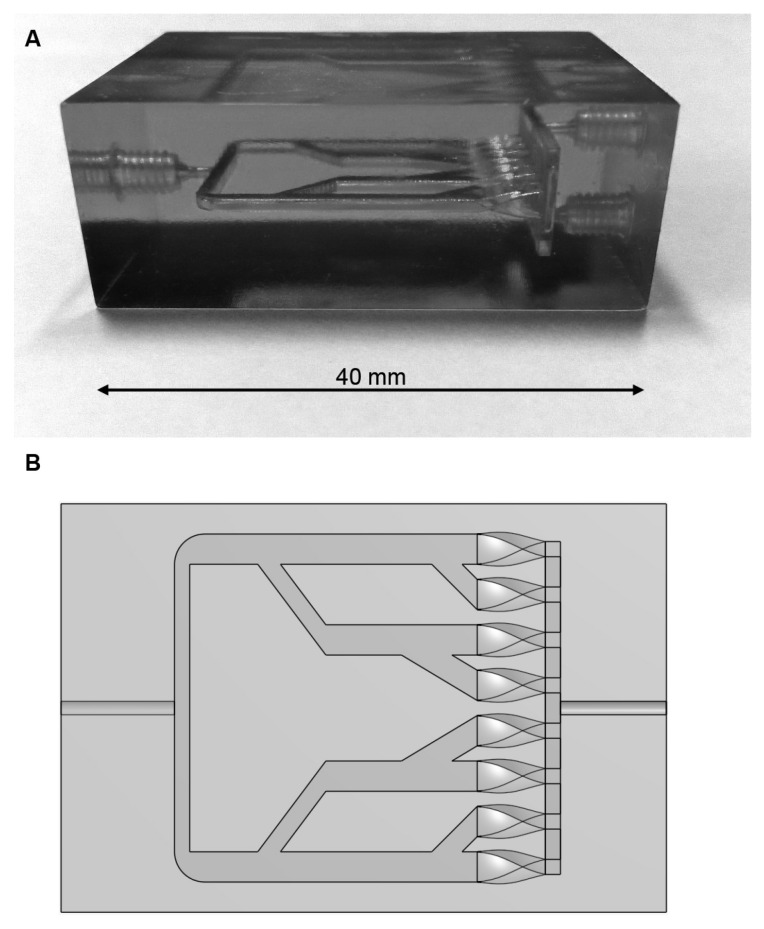
(**A**) Custom built 3D-printed plexiglass mixing device. (**B**) Technical drawing of the mixing device.

A custom mixing cell was developed, as a thorough analysis of the analyzers fluidics had revealed that the fluidics flow resistance was on the upper limit of the pumps’ specifications. The traditional mixing coil (a long piece of coiled tubing) was then replaced by the 3D-printed PMMA mixing cell (Figure 2). The mixer design is based on a multilaminar concept, expanding the contact surface of the two fluid streams by splitting them up in eight streams before combining them again. A CFD (computational fluid dynamics) simulation was used to optimize the design for thorough mixing, low fluidic resistance, and low dead volume. The latter is a disadvantage of any mixer design compared to the mixing coil, as it cannot be completely avoided. The tests of the mixer in the analyzer (e.g., as shown in Section 4.1 when switching between blank and standard solutions) showed a sufficiently fast response, so that the new mixer design was used for operation.

### 2.4. Reagents

**Figure 3:**
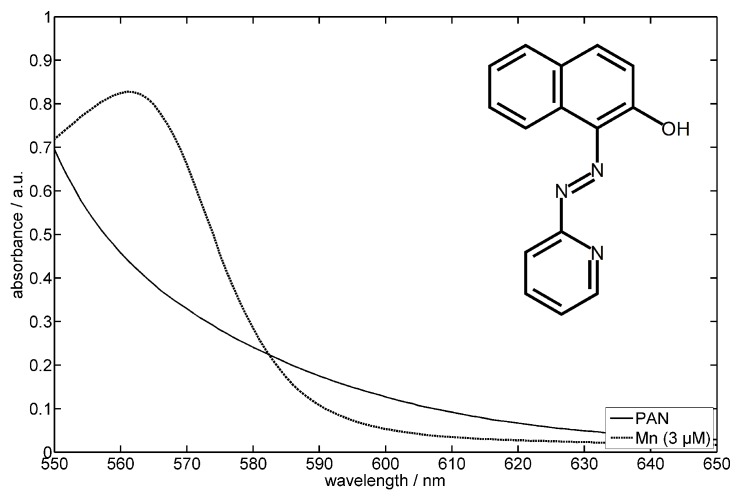
Molecular structure of 1-(2-pyridylazo)-2-naphthol (PAN) as well as absorption spectra of the PAN reagent and the Mn${}^{II}$–(PAN)${}_{2}$ complex.

All reagents used in this study were of analytical grade. The method uses 1-(2-pyridylazo)-2- naphthol (PAN) as spectrophotometric reagent in the presence of Triton X-100 and desferrioxamine B (DFO-B). Figure 3 shows the molecular structure of PAN as well as the absorption spectra of PAN and the Mn${}^{\left(II\right)}$–(PAN)${}_{2}$ complex. PAN is a widely used azo dye, and can be used for the determination of a wide variety of metal ions [48,49]; thus, the selectivity of PAN must be enhanced by the use of a masking agent (DFO-B) and pH control [49,50].

Reagent solution was prepared as follows: (1) 0.2 g of PAN (Sigma-Aldrich Chemie GmbH, Schnelldorf, Germany) and 30 mL of the non-ionic surfactant Triton X-100 (Sigma-Aldrich Chemie GmbH, Schnelldorf, Germany) were added to 500 mL Milli-Q water, and were heated for 2 h at 90 ${}^{\circ }$C under stirring in order to achieve complete dissolution of the hardly soluble PAN [51]; (2) 400 mL of a buffer solution containing NaOH (0.5 M, Merck, Darmstadt, Germany) and $H_{3}{\mathrm{BO}}_{3}$ (0.5 M, Merck, Darmstadt, Germany) were added for pH control; (3) 2 mL of a DFO-B solution (0.25 M, Novartis AG, Basel, Switzerland) was added to mask Fe during sample measurements, which is the only significant interferent to Mn determination in seawater [33]. The resulting solution was then topped up with Milli-Q water to a total volume of 1 L. Due to the formation of polyborates [52] the final reagent solution showed a higher pH (10) as could be expected from the dissociation constant of boric acid (pK${}_{S}$ = 9.25). However, mixing of the reagent with the sample solution at a ratio of 1:1 resulted in a pH of 9.8, where the Mn–PAN complex shows maximum absorbance at the used peak emission wavelength of 562 nm [33]. For the preparation of the standard solutions, an atomic absorption spectroscopy (AAS) standard ($c_{\mathrm{Mn}(\mathrm{II})}$ = 18.2 mM; Merck, Darmstadt, Germany) was used. A blank and a standard solution ($c_{\mathrm{Mn}(\mathrm{II})}$ = 7.5 μM) for the in situ calibration were prepared using sea water from the study area (∼180 m depth). Owing to the hypoxic conditions found at this depth, it was assumed that the dissolved Mn concentrations were negligible (i.e., below the limit of detection of the analyzer, ≤77 nM). In contrast, the blank and the standard solutions for the laboratory calibration were prepared using Milli-Q water to which NaCl was added to achieve a salinity of 10 in order to simulate the ionic strength conditions of the subhalocline waters of the Gotland Basin. This is necessary, as the salinity effect was found to be the only significant matrix effect using the PAN method [53]. Dissolved Mn concentration of the blank solution was determined by ICP-OES, and was below the limit of detection of the method (≤0.8 nM).

### 2.5. Chemical and Physical Parameters

Salinity, temperature, pressure, and dissolved O${}_{2}$ were measured in situ using a CTD-probe (Model SBE-9, Sea-Bird Electronics, Inc., Bellevue, WA, USA). In addition, 66 water samples were collected between 70 m and 200 m water depth (every 2 m) using a pump-CTD system equipped with Teflon tubing to eliminate metal contamination [54], and were analyzed in the home laboratory for total dissolved Mn by ICP-OES in order to validate METIS results. All plasticware was pre-cleaned with 2 vol % sub-boiled HNO${}_{3}$. The water samples were filtered (0.45 μm, SFCA syringe filters, Thermo Fisher Scientific, Waltham, MA, USA), acidified to pH 2 with 65% *v*/*v* sub-boiled HNO${}_{3}$ and stored cool in 2 mL reaction tubes. Total dissolved Mn concentrations were determined by ICP-OES (iCAP 6300 Duo, Thermo Fisher Scientific, Waltham, MA, USA) in axial mode using Sc as an internal standard to eliminate matrix effects. External calibration was carried out with spiked Atlantic seawater (OSIL) diluted to the salinity of the study area. Precision and accuracy of ICP-OES measurements were checked with spiked CASS-5 solutions (National Research Council Canada), and were 5.9% and −5.8%, respectively (n = 25). Finally, the detection limit for dissolved Mn was 0.8 nM.

Furthermore, 29 of the collected water samples between 76 m and 144 m water depth were analyzed for H${}_{2}$S by using the methylene blue method after Cline [55]. Two millilitres of each sample were fixed with 20 μL of a 20 vol % Zn-acetate solution, stored cool, and analyzed using a Specord 40 (Analytik Jena AG, Jena, Germany) spectrophotometer.

## 3. Study Area

In situ experiments of the PAN method with the METIS analyzer were carried out during a cruise with R/V “Poseidon” in the Eastern Gotland Basin (Baltic Sea) in October 2015 (57${}^{\circ }$19′ N; 20${}^{\circ }$03′ E). The Gotland Basin is the largest basin of the central Baltic Sea, and is characterized by a thermohaline stratification [17,56]. Below the steep halocline at about 70 m depth the water is hypoxic; below 100 m depth, anoxic or even euxenic during stagnation periods. This is due to the stratification suppressing deep convection, but also due to the limited lateral deep water ventilation, which is caused by the sills and the narrow and shallow straits connecting the Baltic Sea with the North Sea [57,58]. Therefore, large anoxic zones can usually be observed in this area, showing relatively high concentrations of dissolved Mn at the micromolar level [10,11,21].

However, the environmental conditions during the METIS deployment were different, and influenced by the impact of a Major Baltic Inflow (MBI) event from December 2014 [59]. This event led to a renewal of bottom waters (below 120 m depth) in the Eastern Gotland Basin during Spring 2015, and ended a long-lasting stagnation period. As a result, the deep water in the study area was not entirely anoxic during the “Poseidon” cruise.

## 4. Results and Discussion

### 4.1. Calibration

**Figure 4:**
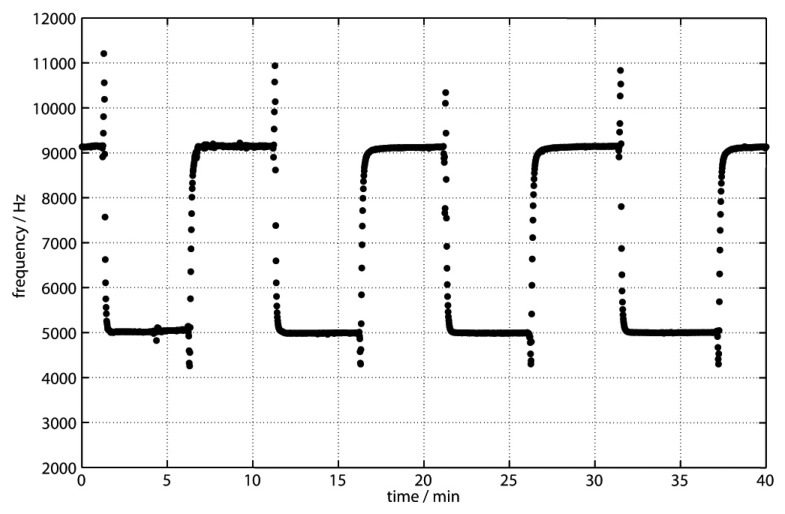
Raw data of the pressure testing tank calibration (2 MPa). Frequency (Hz) as a function of time (min) during the measurement of the blank (higher frequencies) and standard (lower frequencies) solution (Mn(II) = 5 μM). Frequencies are proportional to the light intensity detected by the light-to-frequency converter.

**Figure 5:**
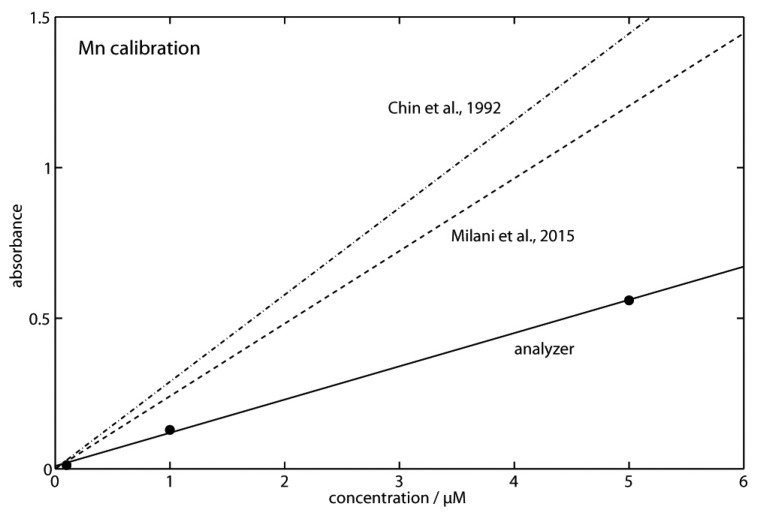
Analyzer calibration lines for dissolved Mn (solid line). Replicate samples (n = 4) were measured for each point. Error bars are within the symbol used for the analyte. The analyzer line is compared to calibrations obtained with other in situ analyzers using the PAN method [33,43]. For comparison purposes, the calibration lines of the other devices are corrected to the analyzer cell length of 14 cm.

Calibration was performed in a pressure test tank at a pressure of 20 MPa (the pressure encountered at a depth of 200 m) using the analyzers’ in situ calibration with different concentrations of standard solution (0.1 μM, 1 μM, and 5 μM). Figure 4 shows one example of the raw data for a standard solution with a concentration of 5 μM. The resulting calibration line for the three standard solutions is shown in Figure 5, and shows a linear response. During the field trials, an experiment was carried out where the analyzer was alternating blank and standard in the same way as in the pressure test tank experiments while it was lowered from 30 m to 200 m depth. No significant pressure effects could be observed.

Identical analyzer parameters were used for calibration and field experiments. The reagent solution was pumped continuously by the reagent pump, which was energized for 300 ms every 1000 ms. The delays of all other pumps relative to the reagent pump were adjusted to 500 ms. They were also energized for 300 ms every 1000 ms when activated. Therefore, the reagent was injected either into the blank or into the standard solution. The sample pump was not used for laboratory calibration.

Although the same chemical method was used, the obtained molar absorption coefficient of $8000\;L\cdot {\mathrm{mol}}^{-1}\cdot {\mathrm{cm}}^{-1}$ is significantly lower than the absorptivities of $17,200\;L\cdot {\mathrm{mol}}^{-1}\cdot {\mathrm{cm}}^{-1}$ [43] and $20,600\;L\cdot {\mathrm{mol}}^{-1}\cdot {\mathrm{cm}}^{-1}$ [33] that were determined by other in situ sensors. This may be due to the high sample-to-reagent ratio of 1:1 chosen for the METIS analyzer in order to ensure sufficient mixing. In contrast, Milani et al. [43] and Chin et al. [33] used sample-to-reagent ratios of 10:1 and 5:1 by using a peak emission wavelength of 572 nm and 569 nm, respectively. Due to this, further developments should include an optimization of this ratio to extend the concentration range and ultimately to enhance the absorption coefficient. Improving the mixing ratio could also help to extend the length of a possible future METIS deployment on an autonomous platform such as a profiling mooring. The current ratio of 1:1 and a 5 L sized plastic bag carrying the reagent solution would limit such a deployment to an amount of 133 Mn profiles, considering a typical profile length of 150 m and a profiling speed of 0.2 m/s. This, for example, corresponds to deployment lengths of 4.5 months and 5.5 days considering a sampling frequency of 1 cast per day and 1 cast per hour, respectively. However, as can be seen from Figure 5, the calibration clearly showed that the METIS system is suitable to provide sufficient sensitivity for Mn analysis in the lower micromolar range. Precision (RSD) values were better than 0.3 %. Linear regression analysis revealed a regression coefficient of R${}^{2}$ = 0.99 with a standard error (SE) of ±0.026. Ultimately, a detection limit (LOD) of 77 nM was calculated (three times standard deviation of the blank signal; n = 4), which is in the range of the LODs reported elsewhere [33,60].

### 4.2. Mn(II) Analysis in the Baltic Sea

**Figure 6:**
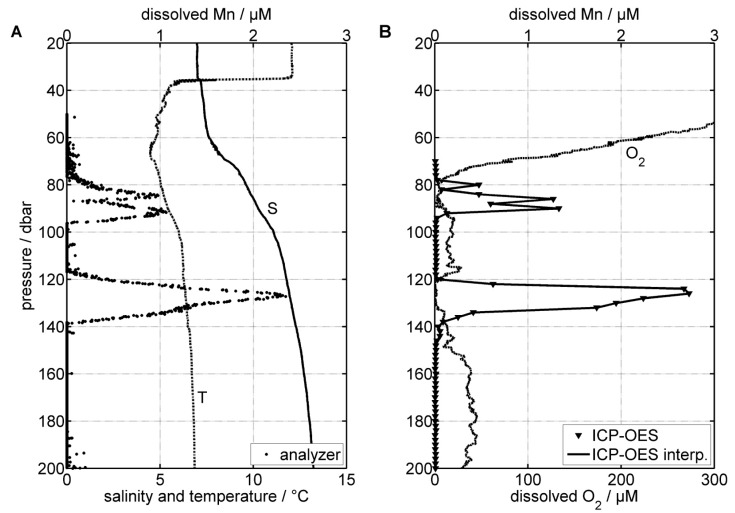
Data from the October 2015 cruise. Vertical profiles of (**A**) temperature, salinity, and dissolved Mn concentrations (METals In Situ analyzer system, METIS); (**B**) dissolved O${}_{2}$ and dissolved Mn concentrations (inductively coupled plasma–optical emission spectrometry, ICP-OES).

Figure 6A shows the vertical profile of dissolved Mn obtained in the central Gotland Basin as well as corresponding temperature and salinity data. The lag time of the analyzer—defined as the time the sample needs from sample intake to the absorption cell—was determined to be t = 8.2 s, and was used for the synchronization with the CTD data. The dissolved Mn(II) concentrations in Figure 6A were calculated using the calibration shown in Figure 5, as the in situ calibration had to be discarded due to malfunctioning blank and standard pumps. The measured frequency values (proportional to light intensities on the detector) between 45 m and 50 m depth were averaged (n = 48) and taken as blank value (f${}_{0}$). Absorbances were then calculated by taking the ratio of f${}_{0}$ and the measured frequency values between 50 m and 200 m depth (f${}_{0}$/f${}_{50}$ to f${}_{0}$/f${}_{200}$). As we took a high resolution vertical profile of bottle samples with the pump-CTD, during this profile a field validation can be carried out by comparing the analyzer results with those of ICP-OES analyses of the bottle samples (Figure 6B).

Field observations of physicochemical parameters show a typical vertical structure of the Gotland Deep, except for dissolved O${}_{2}$ concentrations, which are slightly increased in the deep water as a consequence of the MBI 2014. The halocline—which can be located between 60 m and 80 m water depth—is characterized by a strong salinity gradient. The brackish water of the upper layer exhibits a salinity of around 7, while the more saline deep water reaches values above 13. A thermocline at around 35 m depth separated the warmer surface water (12 ${}^{\circ }$C) and the former winter water (4.5 ${}^{\circ }$C) found beneath 40 m depth. Temperatures of the deep water were found to be relatively low and fairly constant around 6.8 ${}^{\circ }$C. Dissolved O${}_{2}$ concentrations in the surface layer were uniform, averaging 312 μM, but then decreased rapidly between 45 m and 80 m depth. Below the halocline, the dissolved O${}_{2}$ remained generally low, and did not exceed 46 μM. At this point, the water column is hypoxic (DO < 2 mL/L; <∼90 μM) and effectively a “dead zone”. Most higher organisms are negatively impacted, and microorganisms dominate ecosystem processes [61]. In addition, two distinctive anoxic (DO < 0.2 mL/L; <10 μM) and non-sulfidic (H${}_{2}$S < 1 μM) layers were identified between 80 m and 200 m depth. One layer was found between 77 m and 90 m depth, and another layer between 119 m and 138 m depth. Anoxic or at least hypoxic conditions below the halocline are ultimately caused by the limited deep water ventilation in connection with degradation processes of organic matter occurring mainly at the sediment surface [62,63].

As reported in previous studies, total dissolved Mn concentrations ranging from 0.005 μM to 0.04 μM in the upper oxic surface waters of the study area were low, but rapidly increased within the pelagic redoxcline, reaching values of up to 20 μM [10,11,21]. In our study, ICP-OES analysis revealed maximum dissolved Mn concentrations of 1.3 μM and 2.7 μM within the first and second anoxic layers, respectively. The analyzer, however, measured maximum dissolved Mn concentrations of 1.0 μM and 2.4 μM, showing an offset of about 0.3 μM. This may be due to a convolution of the peak signals and the low pass response of the analyzer (e.g., due to residual dead volumes in the mixer). Indeed, the analyzer peaks are broader than the ICP-OES signals, and one small peak at 80 m depth is completely missing, actually indicating that concentration gradients existing between the different samples are partly compensated. In addition, dispersion effects may also be a reason for this compensation, particularly the heterogeneous distribution of the flow velocity within the tubing. Therefore, further developments should aim at reducing the length of tubing between sample inlet and absorption cell in order to lower the lag time and ultimately to limit possible dispersion effects. Another explanation for the differences between the analyzer and the ICP-OES profile could be the sensitivity of the analyzer. It is very likely that the sensitivity of the analyzer decreased between the on-shore laboratory calibration and the reported depth profile of dissolved Mn. Experience has shown that the stability of the sensor baseline varies. An accurate in situ calibration could help to monitor the live performance of the analyzer in future deployments. Furthermore, using sodium dodecyl sulphate (SDS) instead of Triton-X 100 for solubilizing PAN may also help resolving this problem by stabilizing PAN-Mn-micelles (M. Skiba and P. J. Statham pers. comm.). However, possible errors due to salinity, temperature, and pressure were minimized by using similar environmental conditions during the test tank calibration. Artifacts due to competing metal ions were also considered as negligible. Indeed, zinc, copper, nickel, and cobalt produce absorbances that are about 50% of the absorbance of Mn [33], but their concentrations in the study area are very low compared with those of Mn [64]. In contrast to this, uncertainties during the ICP-OES measurements (e.g., analytical error, sample preparation) cannot be excluded. However, although the analyzer values did not match ICP-OES values exactly, it can be seen from Figure 6 that the general shape of the analyzer profile agrees with the corresponding ICP-OES concentrations.

**Figure 7:**
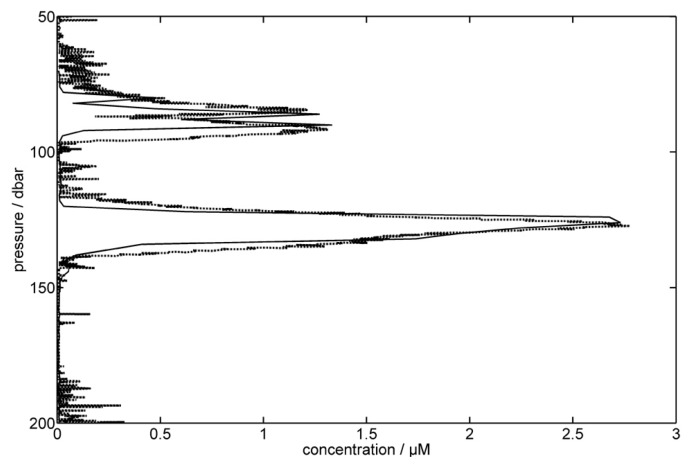
Comparison of bottle measured ICP-OES data (solid line) and METIS results (dashed line) after using ICP-OES concentrations to calibrate METIS absorbances.

**Figure 8:**
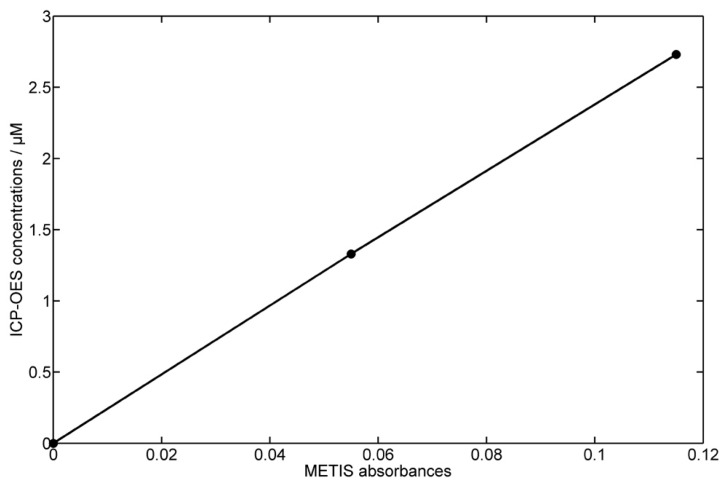
METIS absorbances as a function of dissolved Mn concentrations measured by ICP-OES. Minimum (0.077 μM) and maxima (1.3 μM, 2.7 μM) of ICP-OES measurements were correlated to corresponding METIS absorbances (R${}^{2}$ = 0.99).

An excellent correlation between the bottle measured data and the analyzer data were obtained by fitting METIS absorbances to the Mn concentrations determined by ICP-OES (Figure 7). As can be seen from Figure 8, the correlation between the minimum (0.077 μM) and maxima (1.3 μM, 2.7 μM) of ICP-OES measurements to corresponding METIS absorbances revealed a highly linear calibration line (R${}^{2}$ = 0.99), which can be used to calculate Mn concentrations of the METIS analyzer. This is a strong argument for using METIS on classical ship-based CTD-systems. The use of METIS in this way on a ship-based CTD system immediately has an impact on the vertical resolution of dissolved Mn profiles, but maintains comparability with previous campaigns relying only on the bottle samples. Continued use in this application scenario will develop experience and allow improvement of the system. The longer term goal is autonomous use relying only on the in situ calibration provided in the instrument and pre- and post-calibrations of the analyzer in the laboratory.

In summary, the results showed that in situ analysis of dissolved Mn is a powerful technique providing high resolution data in near real-time without being dependent on heavy and expensive equipment such as a pump-CTD system. When METIS is used on a classical CTD-system, only a few samples for subsequent laboratory analysis are needed to assess the concentration distribution of Mn on a small spatial scale. Furthermore, an autonomous underwater vehicle (AUV) or a long-term moored profiling instrumentation on which a METIS analyzer is attached may help to improve our understanding of Mn cycling in the future. In this case, METIS data would be associated with chemical and physical parameters, helping to investigate mixing processes and reaction kinetics at pelagic redoxclines. This information is required to assess the relevance, for instance, of lateral intrusions of oxygenated waters, vertical eddy-diffusion, and fluctuations caused by internal waves on the Mn cycle. This is particularly important, as horizontal transport appears to play a dominant role along isopycnal surfaces, especially in medium-sized stratified basins [65,66], and yet has not been extensively investigated in the Baltic Sea with respect to the Mn cycle.

## 5. Conclusions

In situ measurements of dissolved Mn carried out with the METIS analyzer were presented. To our knowledge, these data were the first obtained by a multi-pumping flow-system using the PAN method for determining dissolved Mn concentrations at low micromolar levels and with high vertical resolution. We have conducted a hydrocast using a wet chemical analyzer deployed on a CTD-rosette frame. 1-(2-pyridylazo)-2-naphthol (PAN) was used as spectrophotometric reagent. During field experiments, high resolution data of dissolved Mn were obtained in near real-time. Ultimately, a detection limit of 77 nM was obtained. Analyzer results were verified by concurrent analyzes of discrete Mn samples by ICP-OES. In summary, METIS allows a detailed sampling of various study sites where natural Mn concentrations are within the working range of the analyzer, such as pelagic redoxclines [67], hydrothermal vent fields [68], and anoxic estauries [69].

## References

[1] Tebo B.M., Bargar J.R., Dick B.G.C.G.J., Murray K.J., Parker D., Verity R., Webb S.M. (2004). Biogenic Manganese Oxides: Properties and Mechanisms of Formation. Annu. Rev. Earth Planet. Sci..

[2] Ho T.Y., Quigg A., Finkel Z.V., Milligan A.J., Wyman K., Falkowski P.G., Morel F.M. (2003). The elemental composition of some marine phytoplankton. J. Phycol..

[3] Crowley J.D., Traynor D.A., Weatherburn D. (2000). Enzymes and proteins containing manganese: An overview. Met. Ions Biol. Syst..

[4] Kehres D.G., Maguire M.E. (2003). Emerging themes in manganese transport, biochemistry and pathogenesis in bacteria. FEMS Microbiol. Rev..

[5] Brand L.E., Sunda W.G., Guillard R.R. (1983). Limitation of marine phytoplankton reproductive rates by zinc, manganese, and iron. Limnol. Oceanogr..

[6] Coale K.H. (1991). Effects of iron, manganese, copper, and zinc enrichments on productivity and biomass in the subarctic Pacific. Limnol. Oceanogr..

[7] Johnson K.S., Coale K.H., Berelson W.M., Gordon R.M. (1996). On the formation of the manganese maximum in the oxygen minimum. Geochim. Cosmochim. Acta.

[8] Luther G.W., Sundby B., Lewis B.L., Brendel P.J., Silverberg N. (1997). Interactions of manganese with the nitrogen cycle: Alternative pathways to dinitrogen. Geochim. Cosmochim. Acta.

[9] Pohl C., Hennings U. (1999). The effect of redox processes on the partitioning of Cd, Pb, Cu and Mn between dissolved and particulate phases in the Baltic Sea. Mar. Chem..

[10] Dellwig O., Leipe T., März C., Glockzin M., Pollehne F., Schnetger B., Yakushev E.V., Böttcher M.E., Brumsack H.J. (2010). A new particulate Mn-Fe-P-shuttle at the redoxcline of anoxic basins. Geochim. Cosmochim. Acta.

[11] Turnewitsch R., Pohl C. (2010). An estimate of the efficiency of the iron- and manganese-driven dissolved inorganic phosphorus trap at an oxic/euxinic water column redoxcline. Glob. Biogeochem. Cycles.

[12] Van Cappellen P., Wang Y. (1996). Cycling of iron and manganese in surface sediments: A general theory for the coupled transport and reaction of carbon, oxygen, nitrogen, sulfur, iron, and manganese. Am. J. Sci..

[13] Huckriede H., Meischner D. (1996). Origin and environment of manganese-rich sediments within black-shale basins. Geochim. Cosmochim. Acta.

[14] Pakhomova S.V., Hall P.O.J., Kononets M.Y., Rozanov A.G., Tengberg A., Vershinin A.V. (2007). Fluxes of iron and manganese across the sediment-water interface under various redox conditions. Mar. Chem..

[15] Dellwig O., Bosselmann K., Kölsch S., Hentscher M., Hinrichs J., Böttcher M.E., Reuter R., Brumsack H.J. (2007). Sources and fate of manganese in a tidal basin of the German Wadden Sea. J. Sea Res..

[16] Overnell J., Brand T., Bourgeois W., Statham P.J. (2002). Manganese Dynamics in the Water Column of the Upper Basin of Loch Etive, a Scottish Fjord. Estuar. Coastal Shelf Sci..

[17] Neretin L.N., Pohl C., Jost G., Leipe T., Pollehne F. (2003). Manganese cycling in the Gotland Deep, Baltic Sea. Mar. Chem..

[18] Schippers A., Neretin L.N., Lavik G., Leipe T., Pollehne F. (2005). Manganese(II) oxidation driven by lateral oxygen intrusions in the western Black Sea. Geochim. Cosmochim. Acta.

[19] Trouwborst R.E., Clement B.G., Tebo B.M., Glazer B.T., Luther G.W. (2006). Soluble Mn(III) in Suboxic Zones. Science.

[20] Yakushev E., Pakhomova S., Sørenson K., Skei J. (2009). Importance of the different manganese species in the formation of water column redox zones: Observations and modeling. Mar. Chem..

[21] Dellwig O., Schnetger B., Brumsack H.J., Grossart H.P., Umlauf L. (2012). Dissolved reactive manganese at pelagic redoxclines (part II): Hydrodynamic conditions for accumulation. J. Mar. Syst..

[22] Schnetger B., Dellwig O. (2012). Dissolved reactive manganese at pelagic redoxclines (part I): A method for determination based on field experiments. J. Mar. Syst..

[23] Webb S.M., Dick G.J., Bargar J.R., Tebo B.M. (2005). Evidence for the presence of Mn(III) intermediates in the bacterial oxidation of Mn(II). Proc. Natl. Acad. Sci. USA.

[24] Nico P.S., Zasoski R.J. (2001). Mn(III) Center Availability as a Rate Controlling Factor in the Oxidation of Phenol and Sulfide on *δ*MnO_2_. Environ. Sci. Technol..

[25] Madison A.S., Tebo B.M., Luther G.W. (2011). Simultaneous determination of soluble manganese (III), manganese (II) and total manganese in natural (pore) waters. Talanta.

[26] Stramma L., Johnson G.C., Sprintall J., Morholz V. (2008). Expanding Oxygen-Minimum Zones in the Tropical Oceans. Science.

[27] Keeling R.F., Körtzinger A., Gruber N. (2009). Ocean Deoxygenation in a Warming World. Annu. Rev. Mar. Sci..

[28] Gruber N. (2011). Warming up, turning sour, losing breath: Ocean biogeochemistry under global change. Philos. Trans. R. Soc. A.

[29] Klinkhammer G.P. (1994). Fiber optic spectrometers for in-situ measurements in the oceans: The ZAPS Probe. Mar. Chem..

[30] Okamura K., Gamo T., Obata H., Nakayama E., Karatani H., Nozaki Y. (1998). Selective and sensitive determination of trace manganese in sea water by flow through technique using luminol-hydrogen peroxide chemiluminescence detection. Anal. Chim. Acta.

[31] Tercier-Waeber M.L., Belmont-Hébert C., Buffle J. (1998). Real-time continuous Mn (II) monitoring in lakes using a novel voltammetric in situ profiling system. Environ. Sci. Technol..

[32] Luther G.W., Glazer B.T., Ma S., Trouwborst R.E., Moore T.S., Metzger E., Kraiya C., Waite T.J., Druschel G., Sundby B. (2008). Use of voltammetric solid-state (micro) electrodes for studying biogeochemical processes: Laboratory measurements to real time measurements with an in situ electrochemical analyzer (ISEA). Mar. Chem..

[33] Chin C.S., Johnson K.S., Coale K.H. (1992). Spectrophotometric determination of dissolved manganese in natural waters with 1-(2-pyridylazo)-2-naphthol: Application to analysis in situ in hydrothermal plumes. Mar. Chem..

[34] Resing J.A., Mottl M.J. (1992). Determination of manganese in seawater using flow injection analysis with on-line preconcentration and spectrophotometric detection. Anal. Chem..

[35] Chin C.S., Coale K.H., Elrod V.A., Johnson K.S. (1994). In situ observations of dissolved iron and manganese in hydrothermal vent plumes, Juan de Fuca Ridge. J. Geophys. Res..

[36] Massoth G.J., Baker E.T., Feely R.A., Lupton J.E., Collier R.W., Gendron J.F., Roe K.K., Maenner S.M., Resing J.A. (1998). Manganese and iron in hydrothermal plumes resulting from the 1996 Gorda Ridge Event. Deep Sea Res. Part II.

[37] Statham P.J., Connelly D.P., German C.R., Brand T., Overnell J.O., Bulukin E., Millard N., McPhail S., Pebody M., Perrett J. (2005). Spatially Complex Distribution of Dissolved Manganese in a Fjord as Revealed by High-Resolution in Situ Sensing Using the Autonomous Underwater Vehicle Autosub. Environ. Sci. Technol..

[38] Doi T., Takano M., Okamura K., Ura T., Gamo T. (2008). In-situ Survey of Nanomolar Manganese in Seawater Using an Autonomous Underwater Vehicle around a Volcanic Crater at Teishi Knoll, Sagami Bay, Japan. J. Oceanogr..

[39] Johnson K.S., Beehler C.L., Sakamoto-Arnold C.M. (1986). A submersible flow analysis system. Anal. Chim. Acta.

[40] Massoth G. (1991). A SUAVE (Submersible System Used to Assess Vented Emissions) approach to plume sensing: The Buoyant Plume Experiment at Cleft segment, Juan de Fuca Ridge and plume exploration along the EPR9-10 N. Eos.

[41] Okamura K., Kimoto H., Saeki K., Ishibashi J., Obata H., Maruo M., Gamo T., Nakayama E., Nozaki Y. (2001). Development of a deep-sea in situ Mn analyzer and its application for hydrothermal plume observation. Mar. Chem..

[42] Luther G.W., Reimers C.E., Nuzzio D.B., Lovalvo D. (1999). In Situ Deployment of Voltammetric, Potentiometric, and Amperometric Microelectrodes from a ROV to Determine Dissolved O_2_, Mn, Fe, S(-2), and pH in Porewaters. Environ. Sci. Technol..

[43] Milani A., Statham P.J., Mowlem M.C., Connelly D.P. (2015). Development and application of a microfluidic in-situ analyzer for dissolved Fe and Mn in natural waters. Talanta.

[44] Okamura K., Hatanaka H., Kimoto H., Suzuki M., Sohrin Y., Nakayama E., Gamo T., Ishibashi J.I. (2004). Development of an in situ manganese analyzer using micro-diaphragm pumps and its application to time-series observations in a hydrothermal field at the Suiyo seamount. Geochem. J..

[45] Prien R.D., Connelly D.P., German C. (2006). In Situ Chemical Analyser for the Determination of Dissolved Fe(II) and Mn(II). EOS Trans. AGU.

[46] Prien R.D., Schulz-Bull D.E. (2016). Technical note: GODESS - A profiling mooring in the Gotland Basin. Ocean Sci..

[47] Liebig J. (1856). Ueber Versilberung und Vergoldung von Glas. Justus Liebigs Annalen der Chemie.

[48] Cheng K.L., Bray R.H. (1955). 1-(2-Pyridylazo)-2-naphthol as a Possible Analytical Reagent. Anal. Chem..

[49] Cheng K.L., Ueno K. (1982). Handbook of Organic Analytical Reagents.

[50] Chiswell B., Rauchle G., Pascoe M. (1990). Spectrophotometric methods for the determination of manganese. Talanta.

[51] Goto K., Taguchi S., Fukue Y., Ohta K., Watanabe H. (1977). Spectrophotometric determination of manganese with 1-(2-pyridylazo)-2-naphthol and a non-ionic surfactant. Talanta.

[52] Mesmer R., Baes C., Sweeton F. (1972). Acidity measurements at elevated temperatures. VI. Boric acid equilibriums. Inorg. Chem..

[53] Feng S., Huang Y., Yuan D., Zhu Y., Zhou T. (2015). Development and application of a shipboard method for spectrophotometric determination of trace dissolved manganese in estuarine and coastal waters. Cont. Shelf Res..

[54] Strady E., Pohl C., Yakushev E.V., Krüger S., Hennings U. (2008). PUMP–CTD-System for trace metal sampling with a high vertical resolution. A test in the Gotland Basin, Baltic Sea. Chemosphere.

[55] Cline J. (1969). Spectrophotometric determination of hydrogen sulfide in natural waters. Limnol. Oceanogr..

[56] Feistel R., Nausch G., Matthaus W., Hagen E. (2003). Temporal and spatial evolution of the Baltic deep water renewal in Spring 2003. Oceanologia.

[57] Matthäus W., Schinke H. (1999). The influence of river runoff on deep water conditions of the Baltic Sea. Hydrobiologia.

[58] Elken J., Matthäus W. (2008). Baltic Sea oceanography. Assessment of Climate Change for the Baltic Sea Basin.

[59] Mohrholz V., Naumann M., Nausch G., Krüger S., Gräwe U. (2015). Fresh oxygen for the Baltic Sea: An exceptional saline inflow after a decade of stagnation. J. Mar. Syst..

[60] Statham P.J., Connelly D.P., German C.R., Bulukin E., Millard N., McPhail S., Pebody M., Perrett J., Squires M., Stevenson P. (2003). Mapping the 3D spatial distribution of dissolved manganese in coastal waters using an in situ analyser and the autonomous underwater vehicle Autosub. Underw. Technol..

[61] Diaz R.J., Rosenberg R. (2008). Spreading Dead Zones and Consequences for Marine Ecosystems. Science.

[62] Piker L., Schmaljohann R., Imhoff J.F. (1998). Dissimilatory sulfate reduction and methane production in Gotland Deep sediments (Baltic Sea) during a transition period from oxic to anoxic bottom water (1993–1996). Aquat. Microbiol. Ecol..

[63] Zillén L., Conley D.J., Andrén T., Andrén E., Björck S. (2008). Past occurrences of hypoxia in the Baltic Sea and the role of climate variability, environmental change and human impact. Earth-Sci. Rev..

[64] Pohl C., Hennings U. (2005). The coupling of long-term trace metal trends to internal trace metal fluxes at the oxic–Anoxic interface in the Gotland Basin (57°19,20′ N; 20°03,00′ E) Baltic Sea. J. Mar. Syst..

[65] Lewis B.L., Landing W.M. (1991). The biogeochemistry of manganese and iron in the Black Sea. Deep-Sea Res..

[66] Holtermann P.L., Umlauf L., Tanhua T., Schmale O., Rehder G., Waniek J.J. (2012). The Baltic Sea Tracer Release Experiment: 1. Mixing rates. J. Geophys. Res..

[67] Tebo B.M. (1991). Manganese (II) oxidation in the suboxic zone of the Black Sea. Deep-Sea Res. Part A.

[68] Stüben D., Stoffers P., Cheminée J.L., Hartmann M., McMurtry G.M., Richnow H.H., Jenisch A., Michaelis W. (1992). Manganese, methane, iron, zinc, and nickel anomalies in hydrothermal plumes from Teahitia and Macdonald volcanoes. Geochim. Cosmochim. Acta.

[69] Lee D.Y., Owens M.S., Doherty M., Eggleston E.M., Hewson I., Crump B.C., Cornwell J.C. (2015). The effects of oxygen transition on community respiration and potential chemoautotrophic production in a seasonally stratified anoxic estuary. Estuaries Coasts.

